# Dr. Cappe, on the Cow-Pock

**Published:** 1801-01

**Authors:** Robert Cappe

**Affiliations:** York


### To Dr. BATTY.

My dear Sir,

YOU will oblige me by inferting the following Letter, on fome irregularities I have obferved in the Cow-pock, in your next Number of the Medical and Phyfical Journal. I fear that want of care in taking the vaccine matter at a proper period, and watching the inoculation through its progrefs, will bring fome temporary difcredit on the new inoculation in this neighbourhood. I have obferved fome fuch inftances with regret, and have heard of more; I therefore think that every effort to awaken a proper attention to this invaluable difcovery, however feeble, may ferve the great purpofe of exterminating the Small-pox. That terrible diforder is now prevailing with uncommon ſeverity in York. Three children have lately been inoculated with Cow-pox matter, who, in a few days after, ſickened from previous infection of the Small-pox, one on the third day, another on the fourth, and the laſt on the fifth. One is dead, the other two are ſtill under the diſeaſe, but with hope of recovery. The Cow-pox veſicle formed in all, but ſoon loſt its characteriſtic appearance; its progreſs ſeemed to be arreſted at the period at which it was overtaken by the Small-pox.

I am ever your's, affectionately,

R. CAPPE.

---

To the Editor of the York Herald.

Sir,

I have lately heard that in ſome inſtances perſons inoculated for the Cow-pox have immediately removed from the obſervation of the perſon who performed the operation: As ſuch want of care may lead to diſappointment and danger, I think the public ſhould be apprized of the neceſſity of attending to the progreſs of inoculation in its earlier ſtages; for it is only during the early period that the true Cow-pox can be diſtinguiſhed from an irregular effect of inoculation which does not afford ſecurity againſt the Small-pox.

I feel more ſtrongly, that the continued attention of perſons well acquainted with the true character of Cow-pox is neceſſary, from having lately watched with minute care an irregular caſe, in which on the fourth day after inoculation a puſtule aroſe; on the fifth day it became turgid and broke; yet, on the ſixth day it had not entirely diſappeared, and on the ſeventh ſome fluid might ſtill have been obtained by preſſure. The puſtule never aſſumed the true character of Cow-pox: it contained a thin, yellow, muddy fluid, like diluted pus; but, at a late period, this inoculation could not be diſtinguiſhed from that which is a known ſecurity againſt the Small-pox. This puſtule would undoubtedly have deceived an inexperienced eye; and if the deſcription of it given by the parents had been relied on, it is probable that the child would generally have been thought ſecure againſt the Small-pox. Such imaginary ſecurity would unjuſtly bring diſcredit on the operation, and might prove fatal. It is ſurpriſing that no diſappointments of this kind have yet happened; none at leaſt have been made public; though irregular caſes, probably, have fallen under the obſervation of many inoculators; and there may have been others among thoſe inſtances, in which the progreſs of the diſeaſe has not been attended to: in the latter inſtance it cannot not be fuppofed that any fubfequent care has been taken to prevent the Small-pox.

What proportion the irregular cafes may bear to the regular is not yet known. Among 115 patients under my own care, I have feen nine irregular cafes. I am difpofed to believe this proportion uncommonly great. The peculiar circumftances in which three of them occurred, I have ftated in your Herald of the 4th of October, with the caution they fuggefted.* Three others certainly arofe from violence done to the inoculated part. Some of the remaining three might be fairly expected, fince it is obferved that one perfon in fixty of all born,—fome ftate it as one in twenty,† is by natural conftitution incapable of receiving the Small-pox, and is faid to be equally exempt from the Cow-pox.

It has been ftated, that when inflammation appears on the inoculated part before the third day, the Cow-pox will not be produced.—This is not invariably true, though an early inflammation, from whatever caufe it may arife, feems often to prevent abforption of the matter, and when it does not prevent the Cow-pox affuming the true character, it fometimes difturbs the progrefs of the difeafe. Children have, at different periods of the inoculation, fcratched or rubbed the incifion:‡ if this happened when the veficle was beginning to rife, or if the whole furface of the veficle was removed, the difeafe loft its true character. When the veficle was not entirely deftroyed, the difeafe became irregular in its progrefs, an efflorefcence appearing earlier than ufual. When the incifion was much rubbed before the period at which a veficle fhould begin to rife, no veficle was formed, but an extenfive circle, of an almoft evanefcent purple, appeared around the incifion, and a foft pafty cruft was formed upon it.

When a veficle was torn at a late period, it fometimes occafioned a fore; but this confequence was often prevented by applications, that coagulating the fluid, which is copioufly difcharged, form a complete defence againft the air.

I may add, as being immediately connected with this part of the fubject, that when matter is taken for inoculation, it fhould be done in the moft delicate manner; for if the veficle be much injured, the eryfipelas inflammation will be rendered fevere.—

* Viz. Not to blend matter from two patients. Inconvenience may arife even from puncturing a veficle with a lancet not perfectly cleaned from matter of another patient.

† Sauvage and Rofen von Rofenftein.

‡ I have feen the danger of injury been done to the incifion by nurfes not fufficiently careful in gathering up a child's fleeve.

 Though

Though the matter do not flow plentifully at firft, on making a flight puncture in the vefìcle, it will, in a little time, come in fufficient quantity to inoculate many perfons. It feems partly fupplied by fecretion; for I have feen more matter obtained in a quarter of an hour than the veficle could be fuppofed to contain. The matter flows flowly, as the veficle is not one uninterrupted cell, but confifts of many minute cells, which have fo imperfect a communication, that they cannot be entirely emptied by a fingle puncture. During the formation of the veficle, minute cells may often be feen arifing feparately, and they fometimes remain diftinct on the margin. At a late period when a cruft is formed, a ring of cluftered cells fometimes rife around it, more tranfparent than the original veficle. Thefe would probably afford matter for inoculation; for Dr Woodville lately obligingly informed me, that, at the requeft of parents, he had twice inoculated on the thirteenth day from patients, in whom the difeafe was fo far advanced, that he could only obtain a little moifture from the margin of the fcab; yet he was fuccefsful. He concludes, that when the local affection is *regular*, matter for inoculation may be obtained from the fourth to the twelfth day. The knowledge of this fact may be ufeful in remote fituations, though inoculation made with matter as foon as it can be obtained is moft certain. Since this was written, I have learnt from a medical friend in London, from whom farther information on this topic may be expected, that matter taken from a vaccine puftule on the fourteenth or fifteenth day did not produce the Cow-pox, but fevere ulceration. As the time is not ftrictly known when the matter affumes this change, it feems right, for the prefent, to reftrict the taking of matter to that period, during which it has been found uniformly mild and efficient. This correfponds, in general, with the fifth, fixth, feventh, and eighth days after inoculation.

From what I have feen, I muft conclude that the period, at which the inflammation begins, does not ferve invariably for difcrimination between the regular Cow-pox and the irregular effect of inoculation, nor does the time the veficle continues. The cafe referred to fhows that its duration alone ought not to be relied on. But no cafe is recorded of the fluid having difappeared within two days from a veficle that had the true character of Cow-pox: the veficle of the fhorteft duration I have feen lafted many days.

The character of Cow-pox can be learnt only by the eye, yet fome difference between the true difeafe and the fpurious effects of inoculation admits of defcription.—The veficle of the true Cow-pox has, in its early period, a milky whitenefs; the fluid it contains is almoft colourlefs, is as clear as water, and does not change into pus:* it hardens into a cruft, or the vefìcle burfting, it is difcharged. Rednefs appears around the inoculated part in the fpurious cafes, but the efflorefcence of true Cow-pox is more yellow or fcarlet;† and feems to the naked eye to lie upon the furface of the fkin, in appearance not much unlike the eruption of meafles or fcarlet fever. In the fpurious cafes, the rednefs is lefs inclined to yellow, is fometimes purplifh, and feems to be under the furface of the fkin. In all effective inoculations there is a deep hardnefs around the vefìcle, that in general begins before the efflorefcence, or at the time of its appearance. A fimilar hardnefs may be felt in the latter period of fome irregular cafes.

The accurate attention to the rife of the vefìcle, and the appearance of the efflorefcence around it, cannot be too ftrongly enforced; for it is only on the regular appearance of the inoculation in thefe ftages that confidence fhould be placed for fecurity againft the Small-pox. Should future experience difcover that fome irregular effects of inoculation afford fecurity againft the Small-pox, yet this injunction is not too ftrict for the prefent.

If the difeafe commence, as is moft ufual, on the third day, and lofe the character of Cow-Pox within fix days from the time of inoculation, I think it fhould not be relied on; for in fome inftances Dr. Woodville inferted matter of Small-pox on the fifth day after inoculation for the Cow-pox, and each difeafe had its courfe. The trial has not yet been made on the fixth, feventh, or eighth days; but Mr. Loy, of Pickering, informs me, that on the ninth, tenth, and eleventh days he found the inoculation with Small-pox matter abortive. If the fact always prove fo, it is probable that the conftitution is fecure on the ninth day, or it becomes fecure after this period, within the time that the variolous matter requires for commencing its action. If the Cow-pox lofes the true character within the firft nine days, it would, I think, be proper to fubject the patient to a fecond inoculation, to afcertain his fecurity againft the Small-pox.

From the refult of Dr. Woodville's experiments, and the well-known fact that the inoculated Small-pox commence about the eighth day after incifion, it is evident, that the inoculation of Cow-pox is not a fecurity againft the Small-pox, when it anticipates the period of commencement of that difeafe only thirteen

* Sometimes at a late period, the margin of the cruft feparates, and a purulent fluid oozes out; but this does not frequently happen.

† The colour is a lefs brilliant fcarlet in cold than in temperate weather. In cold weather it is proper to clothe the inoculated arm rather warmer than ufual.

thirteen days; and, therefore, as it is well afcertained that the infection of the Small-pox received cafually produces the difeafe about the twefth day, the inoculation of the Cow-pox ought not to be relied on for fecurity, in any inftance, in which a perfon has been previoufly expofed to the effluvia of Small-pox. If the ninth day after inoculation be the earlieft period at which the body becomes fecure through the Cow-pox, then, to give fecurity, the Cow-pox muft anticipate the period of commencement of the Small-pox by feventeen days: And a perfon inoculated with Cow-pox will be liable to injury from effluvia of Small-pox for five days. If fimilar trials be made on the fixth, feventh, and eighth days, it may be determined exactly how long after inoculation for the Cow-pox, a perfon may be injured by expofure to the Small-pox.* But as the Small-pox fometimes commence a little earlier, or a little later than the period mentioned, this time may occafionally vary. Thefe obfervations are ftated merely as fuggefting rules ufeful for the prefent: future experience will probably fhew that they need correction.

Irregularities in the courfe of inoculation is not obferved in the Cow-pox alone; inftances have fometimes occurred of irregular effects from inoculation for the Small-pox, which were erroneoufly fuppofed to afford fecurity againft future infection. I tranfcribe the following as they are related by a medical practitioner of great accuracy and high reputation:—"Laft Spring (1772) I inoculated two children in one family: on the third day there was a flight inflammation around the places of incifion; on the fifth it was confiderably increafed, and the places felt hard upon being preffed by the finger. I faw them again on the feventh or eighth day, and then the inflammation was much increafed, extending nearly to the breadth of half-a-crown. Upon my applying a gentle preffure to the inoculated places, matter iffued out of them; with which, as it iffued from the arms of both patients, I perfectly faturated a cotton thread. With this thread I inoculated nineteen perfons; every one of them had a fever, and eruptions of puftules at a proper time. But the children, from whom the matter was taken, did not ficken as was expected; and, on the eleventh day, the inflammation upon their arms was confiderably abated; and two or three days after this, there remained nothing but a dry fcab. Agreeably to the general opinion of the faculty, I told the parents, that their children were fecure from future infection of

* If thefe fpeculations may be relied on, do they not offer the ftrongeft motive to early inoculation for the Cow-pox, fince we cannot fly to that operation for fafety when the hour of danger is arrived?

of the Small-pox. They, however, infifted upon their being inoculated again, which was accordingly done in the arm of each.—Contrary to my expectation, their arms began again to be inflamed, and went on in the fame manner as they had done before, till about the ninth or tenth day; when they fickened, had a fmart fever for three days, and then an eruption of a confiderable number of variolous puftules."*

If it will not intrude too far on your paper, I requeft you to add the declaration of many eminent Phyficians and Surgeons in London, publifhed laft Auguft, as it may be unknown to fome of your Readers. It is a very ample teftimony to the truth of the facts which have been laid before the public in your paper. If you cannot with convenience infert it this week, I recommend it to your attention at fome future opportunity. I am,

* Mr. Dawfon's Paper in Tranfactions of College of Phyficians in London, vol. 3. p. 385. See eight fimilar cafes related by Mr. Kite in the Memoirs of the Medical Society, London.

